# Climate-driven divergence in plant-microbiome interactions generates range-wide variation in bud break phenology

**DOI:** 10.1038/s42003-021-02244-5

**Published:** 2021-06-16

**Authors:** Ian M. Ware, Michael E. Van Nuland, Zamin K. Yang, Christopher W. Schadt, Jennifer A. Schweitzer, Joseph K. Bailey

**Affiliations:** 1 Department of Ecology and Evolutionary Biology, University of Tennessee, Knoxville, TN USA; 2 Biosciences Division, Oak Ridge National Laboratory, Oak Ridge, TN USA; 3 Department of Microbiology, University of Tennessee, Knoxville, TN USA; 4 Present Address: Pacific Southwest Research Station, Institute of Pacific Islands Forestry, USDA Forest Service, Hilo, HI USA; 5 Present Address: Department of Biology, Stanford University, Stanford, CA USA

**Keywords:** Phenology, Microbial ecology

## Abstract

Soil microbiomes are rapidly becoming known as an important driver of plant phenotypic variation and may mediate plant responses to environmental factors. However, integrating spatial scales relevant to climate change with plant intraspecific genetic variation and soil microbial ecology is difficult, making studies of broad inference rare. Here we hypothesize and show: 1) the degree to which tree genotypes condition their soil microbiomes varies by population across the geographic distribution of a widespread riparian tree, *Populus angustifolia*; 2) geographic dissimilarity in soil microbiomes among populations is influenced by both abiotic and biotic environmental variation; and 3) soil microbiomes that vary in response to abiotic and biotic factors can change plant foliar phenology. We show soil microbiomes respond to intraspecific variation at the tree genotype and population level, and geographic variation in soil characteristics and climate. Using a fully reciprocal plant population by soil location feedback experiment, we identified a climate-based soil microbiome effect that advanced and delayed bud break phenology by approximately 10 days. These results demonstrate a landscape-level feedback between tree populations and associated soil microbial communities and suggest soil microbes may play important roles in mediating and buffering bud break phenology with climate warming, with whole ecosystem implications.

## Abstract (web summary)

Ian Ware et al. use reciprocal plant population by soil location feedback experiments to show how the soil microbiomes of the narrowleaf cottonwood are influenced by genetic and environmental variation, and how these factors affect foliar phenology. They find a landscape-level feedback between tree populations and their associated soil microbial counterparts. This study contributes to the understanding of the interplay between soil, climate, plant and microbial populations with climate warming.

## Introduction

At large geographic scales, understanding how abiotic and biotic factors alter plant phenological traits (i.e., the timing of life cycle events such as leaf bud break) is important for predicting species, community, and ecosystem responses to global change^1–3^. Bud break phenology in temperate deciduous trees is a complex trait widely considered to be driven by the interaction of temperature, photoperiod, and plant genetic architecture^4–6^. Spring leaf-out initiates the growing season and represents a major driver of productivity, resource acquisition, and carbon dynamics, and is thus a critical trait that has far-reaching consequences on the whole ecosystem^7,8^. Alterations to spring bud break can cause asynchrony in associated community interactions, directly influencing food web dynamics and even spatiotemporal patterns in species migration^9–11^. However, in addition to temperature, photoperiod, and plant genetic architecture, recent controlled studies have linked soil microbial communities with changes in phenological variation^12,13^, revealing the surprising importance of biotic interactions in determining phenological events.

Rhizosphere soil microbes can shift plant phenological traits, such as the timing of flowering and leaf-out, leaf longevity, and nutrient acquisition^14–16^, as well as mediate plant growth and fitness^17–20^, highlighting the magnitude of biotic regulation of plant performance and life history events. For example, Wagner et al.^12^ have shown that selection intensity on flowering time in *Boechera stricta* varies depending on the soil microbiome and on abiotic factors as well. Similarly, Lu et al.^13^ mechanistically determined that microbially derived indole acetic acid production delayed flowering in *Arabidopsis thaliana* by downregulating genes responsible for flowering. In addition to microbially mediated phenological changes, microbial symbionts and whole communities have been shown to confer tolerance and fitness advantages to environmental stress^18,21,22^. Gehring et al.^22^, e.g., found that growth in drought-tolerant and drought-intolerant *Pinus edulis* seedlings was similar when given sterile ectomycorrhizal fungi (EMF) inoculum but that drought-tolerant seedlings grew 25% larger than drought-intolerant seedlings under dry conditions when seedlings were allowed to develop in their own distinct EMF communities. Cumulatively, these studies highlight a few of the many roles soil microbial communities play in determining plant phenotypes and plant responses to climatic stressors. However, the roles and subsequent effects of the soil microbiome on plant phenological events across large spatial scale gradients in plant population genetic variation and environmental conditions remain largely unknown^23,24^.

To improve understanding of how plant–microbiome–environment interactions may alter the timing of spring phenology, we combined field observations and a reciprocal population-level greenhouse soil inoculation experiment using soil from across the geographic range of a keystone riparian tree species. In previous research, we showed that climate-driven evolution in bud break phenology created population-level differences in the degree trees condition soil microbial communities and nutrient pools across the range of *Populus angustifolia*^25^ (i.e., plant phenotypes influenced the soil microbiome). In this study, we use modern genomic sequencing and community-level analysis to further examine the interactions between trees and their soil microbiomes with improved resolution. We also experimentally determined whether tree-conditioned soil microbiomes from warm and cool sites differentially influence bud break phenology (i.e., the soil microbiome alters plant phenotype) across a species range. Our overarching hypothesis is that soil microbial communities vary across the geographic range of *P. angustifolia* along strong environmental gradients, and that this variation predictably alters plant phenology in a reciprocal inoculation experiment. Specifically, we hypothesize the following: (1) the degree tree genotypes condition their soil microbiomes varies by population; (2) geographic dissimilarity in soil microbiomes among populations is influenced by both abiotic and biotic environmental variation; and (3) soil microbiomes that vary in response to abiotic and biotic factors can change plant foliar phenology. Consistent with these hypotheses, we show that soil microbiomes vary along a geographic climate gradient, respond to intraspecific variation at the tree genotype and population level, and experimentally show that this variation influences leaf bud break phenology, all of which can have large consequences for ecosystem productivity.

## Results

### Soil microbiomes vary in response to trees, climate, and soil chemistry

**Fig. 1:**
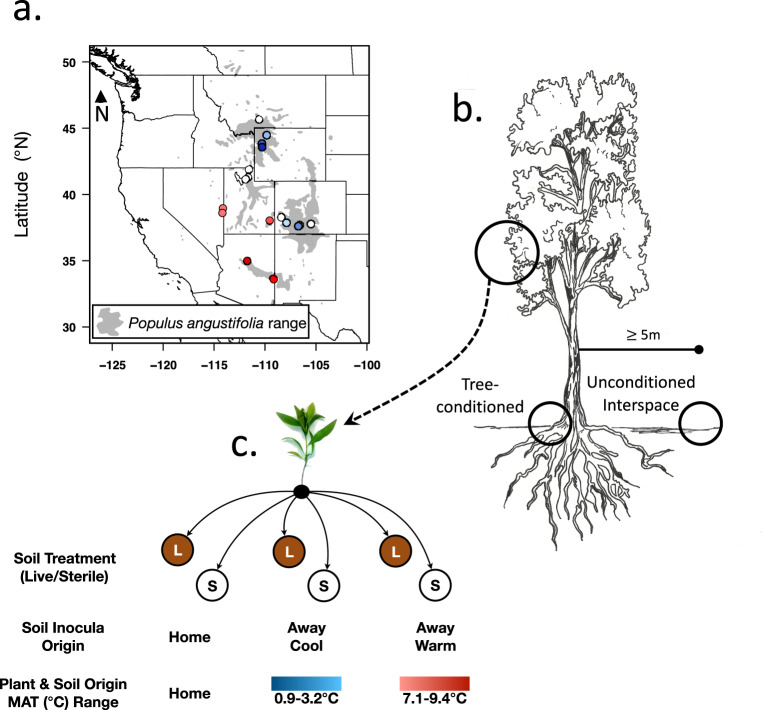
Map of *P. angustifolia* collections and soil inoculation experimental design. **a** Idealized distribution of *P. angustifolia* and collection sites where interspace and tree-conditioned soils and tree genotypes were collected for study. Red symbols represent the five warm sites and blue symbols represent the five cool sites where soils were recollected in 2015 for the soil inoculation experiment. Soil samples for microbial sequencing were collected from all sites (red, blue, and white) in 2012. **b**, **c** 2012 and 2015 field sampling schematic and outline greenhouse soil inoculation experiment to test the hypothesis that soil microbes may mediate variation in bud break phenology. Colored gradient bars match the map in **a** and represent variation in mean annual temperature within five warmest and five coolest tree populations.

**Fig. 2:**
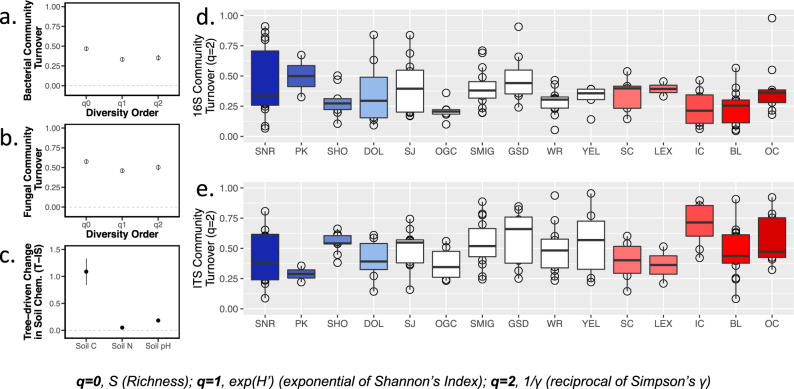
Tree-driven differences in soil microbial communities and soil chemistry. **a** Mean bacterial community turnover for diversity orders *q* = 0–2 (when turnover/β-diversity = 0 tree and interspace communities are identical; when turnover/β-diversity = 1 tree and interspace communities did not share any members). **b** Mean fungal community turnover for diversity orders *q* = 0–2. **c** Mean differences in soil carbon (C), soil nitrogen (N), and soil pH between each tree-interspace pair. Error bars in each panel represent 95% confidence interval of the mean. **d** Population-level differences in bacterial community turnover (*q* = 2; 1/*γ* (reciprocal of Simpson’s *γ*)) between tree-interspace pairs. **e** Population-level differences in fungal community turnover (*q* = 2) between tree-interspace pairs. *P. angustifolia* populations across the 15 watersheds are arranged from coolest to warmest. Center line in each boxplot represents the median turnover for each tree population, end lines represent lower and upper quartiles, and whiskers represent the minimum and maximum turnover estimates within each tree population. Blue and red boxplots match Fig. 1 and represent populations re-sampled in 2015 for soil inoculation experiment. *The soil microbiome was not sequenced for the Gros Ventre River population but was included in the inoculation experiment.

We examined soil microbial communities associated with trees and adjacent interspace soils—to separate the environmental effects from tree-driven conditioning on soil communities—from 15 populations between Arizona and Montana (please see Fig. 1 and Supplemental Table 6 for population descriptions). We identified a total of 3486 bacterial amplicon sequence variants (ASVs) and 2523 fungal ASVs. These ASV’s contributed to soil microbial community composition, taxonomic variation, and species turnover associated with tree-conditioned and adjacent interspace soils across the landscape. Bacterial ASVs could be assigned to 18 phyla, 50 classes, 76 orders, 126 families, and 279 genera (Supplemental Data 1), and fungal ASVs could be assigned to 10 phyla, 30 classes, 63 orders, 108 families, 178 genera, and 215 species (Supplemental Data 2). Overall, our results are consistent with the hypothesis that soil microbiomes respond to tree conditioning across all abiotic conditions, relative to interspace soils not associated with trees (Hypothesis 1). Independent one-sample *t*-tests indicated that three separate metrics of soil microbial community turnover (**q** = **0**, S (Richness); **q** = **1**, exp(H’) (exponential of Shannon’s Entropy Index); **q** = **2**, 1/*γ* (reciprocal of Simpson’s Concentration Index *γ*) between tree and interspace soils across the geographic extent of the study was significantly different than 0 for both soil bacteria and soil fungal communities (Fig. 2a, b, Supplemental Table 1, and Supplemental Data 3 and 4). A turnover = 0 would mean that tree and interspace communities were identical, and a turnover = 1 would mean tree and interspace communities did not share any community members. Observed differences in community turnover suggest that soil bacterial communities underneath trees are on average 46% and 33% different for richness (*q* = 0) and estimates accounting for rare members (*q* = 2), respectively (Fig. 2a). Soil fungal communities underneath trees are on average 57% and 46% different for richness (*q* = 0) and estimates accounting for rare members (*q* = 2), respectively (Fig. 2b). Total soil carbon (C), total soil nitrogen (N), and soil pH also varied between paired tree and interspace soil samples (Fig. 2c and Supplemental Table 2). We also show that the soil microbiome response to trees varied by population, indicating that the soil microbial community turnover in response to tree conditioning varied across the geographic distribution and genetic composition of *P. angustifolia* (Fig. 2c, Supplemental Table 3, and Supplemental Data 5). As the soil microbiome varies in response to tree conditioning locally and across sites, these results provide a foundation for further studies separating the biotic and abiotic factors that drive soil microbiome structure and function across these landscapes in the western United States.

**Fig. 3:**
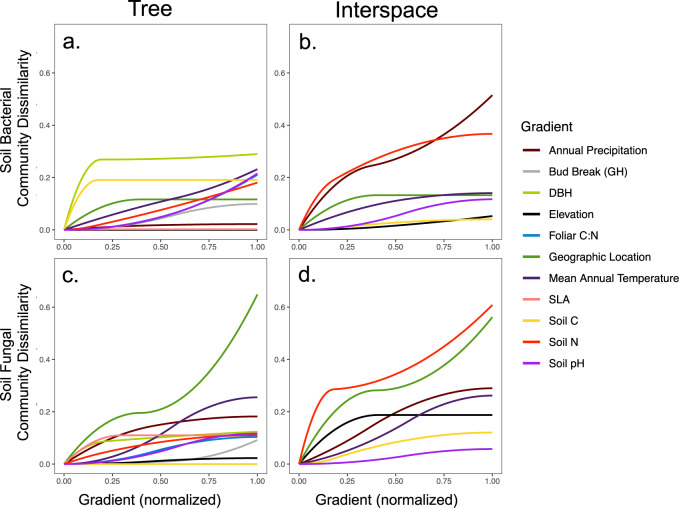
Landscape-level variation in climate, edaphic characteristics, and plant phenotypes drive turnover in soil bacterial and fungal communities. **a** The plotted generalized dissimilarity model (GDM) for tree soil bacterial community dissimilarity. **b** The plotted GDM for interspace soil bacterial community dissimilarity. **c** The plotted GDM for tree soil fungal community dissimilarity. **d** The plotted GDM for interspace soil fungal community dissimilarity. X-axes are normalized to allow for direct comparison of biotic and abiotic environmental gradients included in the analysis.

We used distance-based redundancy analysis (dbRDA) to test the hypothesis that tree-associated soil microbiomes were responding to different environmental factors relative to interspace soil microbiomes across the range of the tree populations (Hypothesis 2). To examine potential biotic environmental drivers of observed variation in the soil microbiome, we measured plant traits in the field and in a common garden. The dbRDA analysis contained data from ~147 replicated genotypes from 15 populations growing under common conditions. Measured field traits included diameter at breast height (DBH), specific leaf area (SLA), and foliar carbon to nitrogen ratios (C : N). In the common garden, we measured bud break phenology, with daily assessments for the date when leaves emerged from the bud, which initiated the growing season. Second, to examine potential abiotic drivers of microbiome community change, we included latitude, longitude, mean annual temperature (MAT), annual precipitation, total soil C, total soil N, and soil pH in our analyses. The dbRDA analysis showed that genetic variation in plant traits and abiotic environmental factors both explain significant variation in tree-associated bacterial and fungal communities (Supplemental Table 4). As expected, there was no relationship between plant traits and interspace soils, as only abiotic factors explained interspace soil microbial community composition. Similar to the dbRDA, generalized dissimilarity models (GDMs) identify significant drivers of community dissimilarity, but also provide additional information on where community dissimilarity changes occur and allow for comparisons among normalized predictors. Our GDMs show that variation in plant phenotypes, tree-associated soil chemistry, and environmental gradients are important in driving community dissimilarity in tree-associated soil bacterial and fungal communities (Fig. 3a, c, Supplemental Table 5, and Supplemental Data 6 and 7). Likewise, geography, interspace soil chemistry, and climate are the most influential drivers of interspace soil bacterial and fungal communities (Fig. 3b, d, Supplemental Table 5, and Supplemental Data 8 and 9). Such consistent patterns and results using powerful multivariate approaches combined with general dissimilarity modeling indicate that: (1) soil microbiomes under trees are different than those in adjacent interspaces and (2) the environmental factors structuring those two community types vary.

To further examine hypothesis 2, we performed redundancy analysis (RDA) to determine whether tree-associated microbial community composition differed among the five warmest and five coolest populations, to examine the potential consequences of warming on the soil microbiome. Based on MAT, the five warmest and five coolest populations span the extremes of the landscape-level temperature gradient and represent climate origin comparisons. We found soil microbial community composition differs for both soil bacteria (bacteria: *χ*^2^ = 166.4, Pr(>*χ*^2^) << 0.0001) and soil fungi (fungi: *χ*^2^ = 4.245, Pr(>*χ*^2^) << 0.00001) between the warm and cool sites regardless of other abiotic variation. Climatic origin RDA models, for both soil bacteria and soil fungi communities, were significantly different than a null model where climatic origin was not included, (bacteria: *p* < 0.001; fungi: *p* = 0.001). Separation and variation in soil microbial community composition among warm and cool habitats is visualized in Supplemental Fig. 1. To provide information on specific taxa and functional groups that reflect the determined differences among the warm and cool site soil microbiomes, we performed indicator species analysis (ISA) on our soil bacterial and fungal community ASV tables. We found large, diverse sets of indicator taxa, of both soil bacteria and fungi, to be strongly associated with either warm or cool habitats (please see Supplemental Data 3–5). From this analysis, we were able to identify 893 bacterial and 553 fungal ASVs significantly associated with cool habitats, and 287 bacterial and 325 fungal ASVs significantly associated with warm habitats (i.e., ASVs assigned a *p* < 0.05). Full indicator species lists and results are displayed in.xls files associated with Supplemental Data 10–12. To gain information on potential functional variation, fungal indicator species lists were analyzed with FUNGuild to assign functional profiles to each fungal assemblage^26^. Of the 325 warm-associated, fungal ASVs, 70 ASVs were assigned to a functional guild with “probable” or “highly probable” confidence rankings. Of the 553 cool-associated, fungal ASVs, 100 ASVs were assigned to a guild with “probable” or “highly probable” confidence rankings. Functional profiles of each fungal assemblage are displayed in Supplemental Fig. 2 and Supplemental Data 11 and 12. These results provide evidence of compositional and functional variation in tree-associated microbiomes between warm and cool populations.

### Role of the tree-associated soil microbiome on bud break phenology

**Fig. 4:**
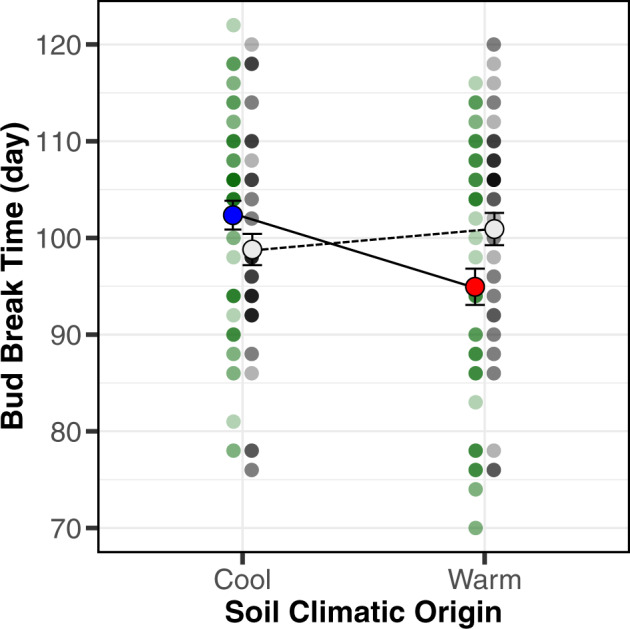
Tree-associated soil microbiomes and their climatic origin mediate bud break phenology. The reaction norm depicts mean soil inoculation effects on bud break phenology (Julian day) across all plant populations when grown in a common environment. Error bars represent ±1 SE from the mean. The blue and red points represent the mean effect of soil inoculations from cool and warm climatic origins, respectively. The white dots represent the mean effect of sterilized inoculations from cool and warm climatic origins. Green and gray dots represent individual data points from live and sterile inoculations, respectively.

We experimentally manipulated soil climatic origin (i.e., soil from warm and cool populations) and soil microbial presence (i.e., live and sterile) to understand how environmentally driven variation in the soil microbiome can change bud break phenology across the geographic range of *P. angustifolia* (Hypothesis 3). In a previous study, we found genetic divergence in bud break phenology in greenhouse common garden conditions, as tree populations from cool locales were breaking bud later than those originating from warm locales^25^. Having previously examined the genetic basis to tree bud break phenology, we then aimed to understand whether tree-conditioned soil microbial communities collected from warm and cool sites have different functional effects on bud break phenology (Supplemental Fig. 3). Consistent with our previous study, there were population-level differences in bud break phenology, irrespective of soil microbiomes (see Supplemental Tables 7 and 8). We also found a significant interaction effect between live/sterile soil microbiomes and soil climatic origin (Supplemental Table 9). As a significant interaction was detected, we used a reduced model including live/sterile as a fixed effect and population as a random effect for both warm and cool soil origin data sets. Adding live microbial inoculations from warm habitats advanced bud break phenology by ~6 days across all populations when compared to sterile inoculations (Fig. 4 and Supplemental Table 9). Further, live microbial inoculations from cool habitats delayed bud break phenology ~4 days, compared to sterile inoculations (Fig. 4, Supplemental Table 9, and Supplemental Data 13). We also examined the effects of soil microbes and soil climatic origin on plant phenology in a continuous framework. We determined the difference between the tree’s climatic origin and the soil inoculum’s climatic origin to provide a “temperature transfer distance.” We show that for every 1° Δ°C in the origin of the live soil treatment there was advancement of bud break phenology in the greenhouse by ~1 day (Supplemental Fig. 4 and Supplemental Table 10). The sterile soil treatment shows no significant pattern (Supplemental Fig. 4 and Supplemental Table 10). Mortality within the experiment was not statistically different among live and sterile inoculation treatments (*χ*^2^ = 0.22, df = 1, *p* = 0.6376). Together, these results provide evidence that soil biota and soil climatic origin (i.e., warm or cool microbiomes) interact to mediate the expression of bud break phenology across large spatial scales and plant host genetic backgrounds.

## Discussion

Our results are consistent with the hypotheses that: (1) soil microbiomes respond to tree conditioning and tree-driven community turnover varies by tree population; (2) geographic variation in tree-associated soil microbiomes are related to biotic and abiotic environmental variation; variation in interspace soil microbiomes is only related to abiotic environmental variation; and (3) experimentally, tree-conditioned microbial communities function differently along a MAT gradient by mediating variation in the timing of bud break in *P. angustifolia*. Together, our findings establish a landscape-level feedback between tree populations and their associated soil microbial counterparts, and suggest that soil microbes may play important roles in mediating and buffering bud break phenology with climate warming.

In this study, we highlighted taxonomic and functional variation in soil microbial communities associated with riparian forests across a large portion of the geographic range of *P. angustifolia*. We find evidence that trees influence their associated soil microbiomes locally and range wide, as suggested by differences in community turnover at both tree and population levels. Determining plant populations condition their soil microbiome differently reinforces the importance of examining the relationship between intraspecific variation and patterns of biodiversity; specifically, in the case presented above, that intraspecific genetic variation can be an important driver of large-scale patterns of soil microbial diversity. Second, we show that both climatic and soil chemistry variation are linked to patterns of soil bacterial and fungal community dissimilarity in interspace soils (Fig. 3 and Supplemental Tables 2 and 4). It is important to note that plant intraspecific variation did not influence interspace soil microbial community variation. In contrast, we identified intraspecific variation in plant phenology, biomass, and foliar chemistry as important drivers of tree-associated bacterial and fungal community dissimilarity, although dominant climate and soil chemistry characteristics were also identified as important (Fig. 3 and Supplemental Tables 2 and 4). Together, these results highlight that intraspecific genetic variation and tree-associated soil microbiomes are responding to similar environmental gradients. Moreover, linkages between intraspecific variation, soil microbial community dissimilarity, and ecosystem characteristics consistently support our previous findings that population divergence and genetic variation alters landscape-level patterns in soil chemistry, soil microbiomes, and eco-evolutionary plant–soil feedbacks^20,25^. On the landscape, plant hosts and associated microbial communities experience similar environmental gradients and may have similar environmental constraints^27^. Similar to plants, there is also evidence that soil microbial diversity, composition, and function can be influenced by climatic, geographic, edaphic, and biotic variation^27–32^. There is also now a large body of evidence that variation in plant community diversity and composition, plant traits, and plant genetics can influence soil microbial diversity and composition^31,33–36^. Although a growing number of studies have linked plant traits and ecosystem characteristics to geographic patterns in soil microbial diversity, few have exclusively linked intraspecific genetic variation to patterns of soil microbial diversity and composition at the landscape level.

We also provide experimental evidence that the geographical variation in the soil microbial community mediated variation in foliar bud break phenology. Adding soil inoculum from either warm or cool populations had the effect of altering bud break phenology by ~10 days when averaged by soil climatic origin (Fig. 4) and up to 16 days when using the temperature transfer distance (Supplemental Fig. 4; *b*^1^ = −0.94; range in soil climatic origin = 17.67 °C). A 10- to 16-day range in microbially mediated phenology corresponds to 36–57% of the total quantitative difference due to genetic variation within *P. angustifolia* (~28 days, based on population-level averages^25^) and is two to three times greater than historical (previous half century) and future model projections of change in temperate tree phenology (5–9.2 days^37,38^). Therefore, plant–microbe interactions could be integral in generating intraspecific variation in response to the climatic variation tree populations experience. The rates of evolutionary response or range shifts in tree species may be outpaced by contemporary (i.e., twentieth century) climatic change^39,40^. However, soil microbes will respond more quickly to changes in climate than tree populations due to differences in generation times and dispersal abilities^41,42^. Therefore, soil microbial mediation of plant phenotypes may represent a mechanism for plant adaptation and persistence, and buffer responses to global change^18,43^.

Mechanistically, ISA and FUNGuild results suggest that the observed change in bud break timing due to the soil microbiome origin could be due to altered species interactions. Our ISA showed a diverse list of soil bacteria and soil fungi under both warm and cool climatic origins (Supplemental Data 3–5). The most apparent difference in warm and cool soil microbial communities from these analyses is the relative frequency of ECM and saprotrophic fungal phyla. We can only speculate what relative differences in the frequency of ectomycorrhizas and saprotrophic fungi could mean in this case. Interactions and contrasts between ECM and saprotrophic fungi have received much attention in regard to soil organic matter decomposition and nutrient cycling^44^, which could certainly influence plant–microbe–soil interactions. We found that warm habitat soils tend to have higher proportion of ectomycorrhizas than cooler soils (Supplemental Fig. 2b), which could suggest a higher probability of plant–fungal symbioses in more stressful environments, as warm sites are warmer and dryer^22,43,45^. In contrast, saprotrophic fungi had a larger proportional frequency in cool soils than in warm soils, and cool habitats are on average much cooler and wetter (Supplemental Fig. 2a). Increasing diversity of saprotrophic fungi may promote the rate of decomposition of soil organic matter^46^ and influence soil nutrient availability. Further exploration of functional variation in soil microbiomes is needed to improve our understanding of direct and indirect consequences of global change scenarios on soil microbial functional variation and related ecosystem functions^47–49^. Likewise, unraveling how plant form and function may be related to soil microbe compositional variation and associated ecosystem processes will yield important context regarding the factors that govern intraspecific genetic variation, patterns of biodiversity, and divergence in ecosystem function in a changing world^50–52^.

Results from this landscape-level field and soil inoculation experiment show plant–soil microbiome feedbacks operate at scales relevant to climate change (i.e., among populations across a species distributional range). Our results demonstrate that soil microbiomes vary in response to the presence of a keystone tree species, and that response varies among tree populations and along ecologically important gradients spanning the western United States. Our results also demonstrate that variation in the soil microbiome due to conditioning by trees can feedback to affect geographic patterns of bud break phenology, which is a critical indicator of the whole ecosystem productivity. Although the soil microbiome is not a panacea, it may provide a 10- to 16-day phenological buffer to climate warming and enhance the ability of plants to respond to global change. Whether or not these results represent a pattern of local adaptation is unclear, but soil microbiomes respond to population-level differences in tree conditioning and trees respond differently to tree-conditioned soil microbiomes across the western United States, a pattern that is consistent with co-adaptation. As important genetically based biotic interactions change in response to climate change, we must continue to examine the role intraspecific variation and associated patterns of biodiversity play in mediating plant phenotypes and variation in ecosystem functions.

## Methods

### Study species and site selection

*P. angustifolia* James is a dominant tree species distributed throughout high elevation riparian zones (900–2500 m) along the Rocky Mountains from southern Alberta, through the intermountain United States, and into northern Mexico^53^. During May and June 2012, 17 distinct *P. angustifolia* populations were surveyed collectively from three different genetic provenances (Arizona, Eastern, and Northern/Wasatch Clusters^54^) across a gradient of ~1700 km latitude from southeastern Arizona to south central Montana. Only 15 of which are used in the observational portion of the study. All trees used in the study were GPS located in the field and 18 bioclimatic traits were determined for the collection sites along each river (via WorldClim^55^) See Ware et al.^25^ for details regarding established greenhouse common garden and methods for climatic data extraction.

### Characterizing soil microbial communities

To understand the range-wide variation in the *P. angustifolia*-associated soil environment, paired conditioned (i.e., tree associated) and unconditioned soils were collected for each tree surveyed across the range of *P. angustifolia* at the same time cuttings were collected. To separate the conditioning effects of *P. angustifolia* from underlying site differences, tree-conditioned soils were collected at the base of each trunk (within 0.25 m) and unconditioned interspace soils were collected from a random location away from the tree canopy, no less than 5 m from the trunk and consistently outside of the drip line of each tree canopy. In 2012, soil samples were collected with a 2.5 cm diameter Oatfield soil core to a vertical depth of 15 cm, placed in a plastic bag, transported cold from the field, and stored at 4 °C in the lab until analysis. Field-collected wet soils were sieved to 2 mm and then sub-sampled and preserved for various analyses^25^. Soil DNA was extracted from a 0.25 g frozen sub-sample of each sieved soil by using the Power Soil DNA isolation kit (MoBio, Carlsbad, CA USA) according to the manufacturer’s instructions. Quantitative PCR reactions to assess bacterial and fungal abundance in each field soil sample were performed after Castro et al.^56^ and Wilson et al.^57^ in 96-well plates on a CFX96 real-time PCR detection system (Bio-Rad Laboratories, Hercules, CA, USA). Samples were amplified for the 16S v4 region using primers 515F/806R, and for the ITS2 region using primers ITS9F/ITS4R from a subset of the total field collections (~270 samples; across 15 populations). Samples were sent to the Department of Energy Joint Genome Institute for sequencing on an Illumina MiSeq (2 × 300 bp; Illumina, Inc., San Diego, CA). The resultant demultiplexed samples underwent initial pre-processing using BBTools. Specifically, adapters were trimmed and contaminants were filtered from reads using BBDuk. Paired-end reads were then merged with BBmerge before further processing.

### Soil inoculation experiment

To address whether plant genetic variation, soil biota, and soil climatic origin interact to influence plant bud break phenology, we established a greenhouse soil inoculation experiment. Replicated tree genotypes were collected in 2012 and grown in a common greenhouse environment. The greenhouse common garden is located at the University of Tennessee in a glass greenhouse allowed to follow seasonal changes in temperature. Saplings grew for 3 years in ambient light with weekly water and monthly fertilizer during growing season for maintenance (a water-soluble, balanced 20 : 20 : 20 of N, P, K). During establishment period (prior to experiment), Ultra‐Pure Oil Horticultural Miticide/Insecticide/Fungicide treatments were applied before bud break, after leaf senescence and as needed to control foliar fungal and pest outbreaks. In May 2015, tree-conditioned soils were recollected from ten of the *P. angustifolia* populations (previously surveyed in 2012) for experimental inoculations (Fig. 1a). Based on MAT, the five warmest and five coolest populations (referred throughout as warm vs. cool or climatic origin comparisons) from the original 2012 survey were included in the inoculation experiment to span the extremes of the landscape-level temperature gradient (see Supplemental Table 6). For each of the ten populations re-sampled, we surveyed five tree genotypes and collected tree-associated soils from each of the five genotypes. Previous work in these same *P. angustifolia* populations shows dominant taxonomic groups of soil microbes did not differ between years from re-sampled populations, indicating that the identity of dominant taxa were generally consistent across years^58^. Half of each soil sample collected was sterilized using γ-irradiation (exposed to a radioisotope cobalt 60 radiation field and irradiated at ~25–30 kGy; STERIS Corporation; Spartanburg, SC), to specifically test the influence of a live soil microbiome on bud break phenology and plant growth traits expressed in the common garden trial. Each tree in the experiment was extracted from their original pots and the soil was carefully removed from their roots. Trees were placed into new pots that were filled to ~80% capacity with general potting mix before receiving soil inoculum. Approximately 20 g of either live or sterile soil inoculum was added directly to the upper portion of each tree’s root system and then covered with general potting mix in an effort to minimize cross-contamination among pots in the experiment. Each replicated genotype was inoculated with live and sterile soil from the site where the tree was collected (hereafter “home” soil), as well as a random soil from a site represented by climate (hereafter referred to as “away” warm or “away” cool soil). For example, one genotype replicate from a warm population was inoculated with live (i.e., microbes present) and another replicate of the same genotype was inoculated with sterile soil from its home soil, live and sterile soil from a random warm population’s soil, and live and sterile soil from a random cool population’s soil. This was replicated for all genotypes and populations (~250 trees survived). Following the 2015 growing season, all trees in the inoculation experiment senesced and entered dormancy. Starting in February 2016 (i.e., the first spring after experiment was established), foliar bud break phenology was measured every 48 h until all trees had flushed. Bud break was recorded as the ordinal day when new leaves unfurl during spring emergence, representing the onset of annual aboveground biomass production^59,60^.

### Statistics and reproducibility

#### Soil microbial community assessments

To address Hypotheses 1, we processed iTags from soils collected in 2012 from beneath trees, paired with an interspace soil, to identify ASVs, using DADA2 version 1.6.0^61^. Reads were truncated to 280 bp to remove low-quality nucleotides at the tails and were quality filtered by removing PhiX contamination and allowing a maximum of 1 expected errors (maxEE = 1). A parametric error model was learned from the data and identical sequences were dereplicated before ASVs were identified and an ASV table (analogous to an operational taxonomic unit (OTU) table) was constructed. This workflow was performed for each of the three plates, resulting in three ASV tables. As DADA2 identifies ASVs (rather than clustering OTUs based on similarity), the three ASV tables were merged into a single table from which chimeras were removed. Taxonomy was assigned for each unique ASV using Ribosomal Database Project (RDP) training set 16 (16S) and the UNITE 28/06/2017 general release (ITS2). In total, 48,686 bacterial/archaeal and 50,630 fungal ASVs were identified from 147 samples. To focus on the most prevalent taxa, we filtered bacterial ASVs not seen more than three times in at least 10% of samples and fungal ASVs not seen more than three times in at least 5% of samples (different filtering criteria used to account for sparser fungal ASV tables). This resulted in a total of 3486 bacterial ASVs and 2523 fungal ASVs that were used to analyze soil microbial community variation and taxonomic composition. Archaea accounted for <1% of the filtered ASVs (26 out of 3486 ASVs). This is expected given the 16S primers the Joint Genome Institute (JGI) used for sequencing are known to underrepresent Archaeal taxa^62^.

To understand whether trees directly influence soil microbial communities (Hypothesis 1), we calculated community turnover (pairwise β-diversity) between tree and interspace samples. Turnover was calculated for both bacterial (16S) and fungal (ITS2) communities using diversity orders *q* = 0–2 [**q** = **0**, *S* (Richness); **q** = **1**, exp(H’) (exponential of Shannon’s Entropy Index); **q** = **2**, 1/*γ* (reciprocal of Simpson’s Concentration Index *γ*)] (i.e., Hill numbers^63–65^). Hill numbers (1) are all expressed in units of effective numbers of species and incorporate relative abundances; (2) provide a direct link with differentiation (i.e, compositional similarity) among assemblages uniting diversity and similarity; (3) account for rare community members as the order of *q* increases; and (4) are being increasingly used to characterize taxonomic diversity within and among assemblages of interest^63,64^. Each tree-interspace pair was rarefied to the sample with the lowest number of reads. Relative abundances for each community member were determined and β-diversity was calculated for each order of *q* (0–2) using the “vegetarian” R package. A *t*-test was used to test whether estimates of turnover differed from 0. A turnover = 0 would mean that tree and interspace communities were identical and a turnover = 1 would mean tree and interspace communities did not share any community members. A generalized linear model was used to explore among population-level variation in both bacterial and fungal community turnover for each order of *q* (glm function).

To identify the environmental drivers of soil microbial community composition at the landscape-scale, we used dbRDA in the vegan R package using soil and climatic data previously reported in Ware et al.^25^. Individual dbRDAs were completed separately for tree-associated bacteria, interspace bacteria, tree-associated fungi, and interspace fungi. Jaccard distance was used to determine dissimilarity among samples and dissimilarity matrices were included in the dbRDA as the response variable. Biotic and abiotic environmental variables were included in each dbRDA model including the following: latitude, longitude, MAT, annual precipitation, total soil C, total soil N, soil pH, field DBH, field SLA, field Foliar C : N, and common garden genetic variation in bud break phenology. Each dbRDA model was analyzed using anova.cca() with resampling (permutations = 10,000) to identify significant environmental predictors. Including field and common garden traits allows for exploring the importance of genetic variation in phenology and productivity in determining soil microbial community composition. The same model was run for each of the four community matrices (i.e., tree-associated bacteria, interspace bacteria, tree-associated fungi, and interspace fungi). This approach allows us to understand if plant traits are of any importance to interspace soil microbial communities, providing further evidence that intraspecific variation among individual trees are conditioning their associated soil microbial community. To compliment dbRDA, we explored soil bacterial and fungal community dissimilarity using GDM^66,67^ (gdm R package). GDM models biological variation as a function of environment and geography using distance matrices—specifically by relating dissimilarity in species composition. The same variables used in the dbRDA were included in individual GDMs for tree-associated bacteria and tree-associated fungi. If plant traits are determined to be unimportant in the dbRDA framework, they will be excluded from GDM models for interspace bacteria and interspace fungi. Similar to dbRDA, the GDMs identify significant drivers of community dissimilarity, but will also provide additional information on where community dissimilarity changes and allows for comparisons among individual predictors.

#### Reciprocal soil microbiome experiment

We developed multiple statistical models to look for patterns of local adaptation or phenotypic plasticity in plant phenological responses to soil microbial inoculations and origin (Hypothesis 2). Four models were constructed to look for potential patterns of local adaptation (i.e., a significant genetic by environment interaction—G × E) to both population-level and soil climatic origin-level variation in soil microbiomes. The first was a fully factorial generalized linear model with tree population (i.e., background tree genetics), soil source population (i.e., the population where the soil inoculum was collected), and their interaction term as fixed effects. The second was a fully factorial generalized linear model with tree population home or away (i.e., whether tree was inoculated with soil from its home population or an away population), and their interaction term as fixed effects. The third was a fully factorial generalized linear model with tree population, soil climatic origin (i.e., climatic origin: warm or cool origin), and their interaction as fixed effects. The fourth was a fully factorial mixed effects model with tree climatic origin (i.e., warm or cool), soil climatic origin (i.e., warm or cool origin), and their interaction as fixed effects, and tree population included as a random effect (lme4 package). Population was included as a random effect, to account for variance in plant traits. A significant interaction term in either of the three models would suggest local adaptation to the soil microbiome at different geographic scales. Each of the four models above was run on both live and sterile data sets to directly test the effect of soil microbial communities on plant phenotypes. Bonferonni corrections were applied to account for multiple comparisons. If no significant genetic × environment effects were discovered, then a fully factorial linear mixed effects model was constructed with live/sterile (i.e., with and without microbes) and soil climatic origin (i.e., from warm or cool habitats), and subsequent interactions were included as fixed effects to explore patterns of phenotypic plasticity. Population was included as a random effect, to account for population-level variance in plant traits. If the full model yields a significant interaction term, the model will be reduced to specifically examine individual fixed effects. We also examined the effects of soil microbes and soil climatic origin of plant phenology in a continuous framework. The difference between the tree’s climatic origin and the soil inoculum’s climatic origin was determined to provide a “temperature transfer distance” and is referred to as Δ°C in any accompanying figure or table. This experimental Δ°C represents a hypothetical, temperature-based change to where the tree is rooted. As soil microbial generation times are likely orders of magnitude quicker than *P. angustifolia* genotypes, this experimental “temperature transfer distance” manipulation serves as a useful tool to examine how warming may indirectly influence the plant phenotypes as climate alters the soil environment in which longer lived plants occupy. Mortality within the experiment was recorded and analyzed using a *χ*^2^-test to determine whether there were non-random effects of live vs. sterile soil inoculation on plant mortality.

To explore compositional differences and provide information on specific taxa and functional groups that reflect the determined differences between warm–cool soil microbiomes implemented in the soil inoculation experiment, we took a two-step approach. First, we performed RDA (vegan R package) on both tree-associated soil bacterial and soil fungal communities to ask whether soil microbial communities differed among warm and cool populations (i.e., climatic origin). The anova.cca() function with resampling (permutations = 10,000) was used to test the significance of climatic origin. Analysis of variance was used to compare each RDA model to a null model. Second, we performed ISA (indicspecies R package) on our tree-associated soil bacterial and fungal community ASV tables, to identify specific taxa and functional groups that were strongly associated with warm or cool tree populations/habitats. Indicator fungal taxa lists were assigned to functional guilds, using an open annotation tool (FUNGuild^26^). Only the guild assignments with “probable” and “highly probable” confidence rankings were accepted. All analyses were performed in R^68^ unless otherwise noted.

### Reporting summary

Further information on research design is available in the Nature Research Reporting Summary linked to this article.

## Supplementary information

Supplemental Information

Description of Additional Supplementary Files

Supplemental Data 1

Supplemental Data 2

Supplemental Data 3

Supplemental Data 4

Supplemental Data 5

Supplemental Data 6

Supplemental Data 7

Supplemental Data 8

Supplemental Data 9

Supplemental Data 10

Supplemental Data 11

Supplemental Data 12

Supplemental Data 13

Reporting Summary

## Data Availability

Amplicon sequences are archived in the National Center for Biotechnology Information SRA database (BioProject accession number: PRJNA726831). Observational and experimental data generated during and/or analyzed during the current study can be found as Supplementary Data 1–13.

## Code availability

All codes were written and performed in R (version 3.5.2). Codes generated during this study are available from the corresponding author upon reasonable request.
