# Supplementary material for: Climate-driven divergence in plant-microbiome interactions generates range-wide variation in bud break phenology
Source: Commun Biol. 2021 Jun 16;4:748. doi: 10.1038/s42003-021-02244-5 (PMC8209103; doi:10.1038/s42003-021-02244-5)
Supplement: Supplementary file 1 — Supplemental Information [file 42003_2021_2244_MOESM1_ESM.docx]

# Title: Climate-driven divergence in plant-microbiome interactions generates range-wide variation in bud break phenology

# Authors:

# Ian M. Ware^1,2*^, Michael E. Van Nuland^2,3^, Zamin K. Yang^4^, Christopher W. Schadt^4,5^, Jennifer A. Schweitzer^2^, & Joseph K. Bailey^2^

Author Affiliations:

^1^Current affiliation: Institute of Pacific Islands Forestry, USDA Forest Service, Pacific Southwest Research Station, Hilo, HI, USA 96720

^2^Department of Ecology and Evolutionary Biology, University of Tennessee, Knoxville, Tennessee, United States of America, 37996

^3^Current affiliation: Department of Biology, Stanford University, Stanford, California, United States of America, 94305

^4^Biosciences Division, Oak Ridge National Laboratory, Oak Ridge, Tennessee, United States of America, 37831

^5^Department of Microbiology, University of Tennessee, Knoxville, Tennessee, United States of America, 37996

*Present Address:

Ian M. Ware, Institute of Pacific Island Forestry, Pacific Southwest Research Station, USDA Forest Service, 60 Nowelo Street, Hilo, HI, 96720, [ianmware@gmail.com](mailto:ianmware@gmail.com), +19013515410

**Supplemental Figures and Legends**

**Supplemental Figure 1. Population-level relative abundances for dominant bacterial and fungal taxa from tree-conditioned soil.** Panels **a.** and **b.** show bacterial and fungal community structure, respectively, varies among the five warmest (in red) and four* coolest (in blue) populations, corresponding to shifts in the relative dominance of major taxa. Panel **c.** shows the relative abundance of dominant bacterial taxa from tree-conditioned soils. Panel **d.** shows the relative abundance of dominant fungal taxa from tree-conditioned soils. Red and blue gradient bars match the map in Figure 1 and represent variation in mean annual temperature within 5 warmest and 5 coolest tree populations. *The soil microbiome was not sequenced for the Gros Ventre River population but was included in the inoculation experiment.

**Supplemental Figure 2. Tree-associated soil fungal guild variation for cool and warm indicator fungi.** Panel **a.** shows the proportional frequency of each fungal guild associated with cool habitats. Panel **b.** shows the proportional frequency of each fungal guild associated with warm habitats. Associated taxa were identified using indicator species analysis, and indicator species lists were analyzed with FUNGuild to identify guilds.

**Supplemental Figure 3. Landscape-level observational relationship between soil microbiome and bud break phenology.** Panel **a.** presents the significant, observational relationship between landscape-level genetic variation of bud break phenology in the greenhouse common garden and the tree-associated soil fungi to bacteria ratio from field collected soil samples (*X*^2^_(1,13)_=4.245, Pr(>*X*^2^)=0.039). In contrast, panel **b.** shows a lack of relationship between interspace soil microbial communities and the genetic cline in bud break phenology. (Pr(>X^2^)=0.864). Blue data points represent trees and soil from cool climatic origins and red data points represent trees and soil from warm climatic origins.

**Supplemental Figure 4. Tree-associated soil microbiomes and temperature transfer distance mediate the timing of bud break phenology.** This figures shows that the difference between tree genotype climatic origin and soil microbiome climatic origin (i.e., temperature transfer distance, $\Delta℃$) predicts variation in the timing of bud break (green line). Dotted black line represents insignificant relationship of sterilized soil transfer distance (i.e., the difference between MAT of soil inoculation origin and MAT of plant origin) and the timing of bud break. Lack of significant relationship with sterilized soil transfer function, provides evidence of a climate-driven microbial mediation of bud break phenology. Green dots represent live inoculations and black dots represent sterile inoculations. Dotted red line represents experimental mean phenology across the entirety of experimental inoculations.

**Supplemental Tables and Legends**

**Supplemental Table 1. Tree-driven conditioning of soil microbial communities.** One-sample t-test results testing the hypothesis that true mean community turnover is greater than zero across diversity orders (q=0-2; **q=0**, S (Richness); **q=1**, exp(H’) (exponential of Shannon’s Entropy Index); **q=2**, 1/γ (reciprocal of Simpson’s Concentration Index γ)).

This table is visually represented in Figure 2a & b.

| **Data** | **DF** | **t** | **Pr(>t)** |
| --- | --- | --- | --- |
| Bacterial mean turnover (q=0) | 128 | 40.401 | <0.0001 |
| Bacterial mean turnover (q=1) | 128 | 27.317 | <0.0001 |
| Bacterial mean turnover (q=2) | 128 | 19.91 | <0.0001 |
| Fungal mean turnover (q=0) | 127 | 50.29 | <0.0001 |
| Fungal mean turnover (q=1) | 127 | 34.91 | <0.0001 |
| Fungal mean turnover (q=2) | 127 | 28.161 | <0.0001 |

**Supplemental Table 2. Tree-driven conditioning of soil chemistry.** One sample t-test results testing the hypothesis that the tree conditioning effect (T-IS) on total soil C, total soil N, and soil pH is greater than zero. Results displayed in Figure 2c.

| **Data** | **DF** | ***t*** | **Pr(>t)** |
| --- | --- | --- | --- |
| Soil C Difference (T-IS) | 573 | 8.71 | <0.0001 |
| Soil N Difference (T-IS) | 567 | 7.32 | <0.0001 |
| Soil pH Difference (T-IS) | 535 | *8.66* | <0.0001 |

**Supplemental Table 3. Population-level variation in bacterial and fungal community turnover.** Generalized linear model results show population-level differences in tree-driven community turnover in soil bacteria and fungi (**q=0**, S (Richness); **q=1**, exp(H’) (exponential of Shannon’s Entropy Index); **q=2**, 1/γ (reciprocal of Simpson’s Concentration Index γ)).

| ***Response:*** | *Bacterial Community Turnover* | | | *Fungal Community Turnover* | | |
| --- | --- | --- | --- | --- | --- | --- |
| **Factor** | **DF** | ***X*^2^** | **Pr(>*X*^2^)** | **DF** | ***X*^2^** | **Pr(>*X*^2^)** |
| Population (q=0) | 14 | 33.2 | **0.002** | 14 | 30.5 | **0.007** |
| Population (q=1) | 14 | 30.8 | **0.005** | 14 | 24.6 | **0.039** |
| Population (q=2) | 14 | 24.8 | **0.03** | 14 | 24.4 | **0.041** |

**Supplemental Table 4. Drivers of soil microbial community composition.** Distance-based redundancy analysis (dbRDA) shows plant phenotypic variation and abiotic environmental variation influence tree-associated soil bacterial and fungal communities. Only abiotic environmental variation explained significant variation in interspace soil bacterial and fungal communities. Significant predictors are in bold. (F) denotes field measured plant traits, and (GH) denotes genetic variation in plant traits measured in the greenhouse common garden. Constrained axes for model predicting tree bacterial community explained 24.7% of the variation in community composition (dbRDA1: 27.5%; dbRDA2: 13%). Constrained axes for model predicting interspace bacterial community explained 24.27% of the variation in community composition (dbRDA1: 26%; dbRDA2: 16%). Constrained axes for model predicting tree fungal community explained 22.99% of the variation in community composition (dbRDA1: 19%; dbRDA2: 12.5%). Constrained axes for model predicting interspace fungal community explained 24.3% of the variation in community composition (dbRDA1: 16.6%; dbRDA2: 13%). All 15 populations represented in Figure 2d & e are included in distance-based redundancy analysis.

| ***Response:*** | *Tree Bacterial Community* | | | *Interspace Bacterial Community* | | | *Tree Fungal Community* | | | *Interspace Fungal Community* | | |
| --- | --- | --- | --- | --- | --- | --- | --- | --- | --- | --- | --- | --- |
| **Factor** | **DF** | ***X*^2^** | **Pr(>*X*^2^)** | **DF** | ***X*^2^** | **Pr(>*X*^2^)** | **DF** | ***X*^2^** | **Pr(>*X*^2^)** | **DF** | ***X*^2^** | **Pr(>*X*^2^)** |
| Latitude | 1 | 7.06 | **0.007** | 1 | 21.8 | **<0.0001** | 1 | 32.47 | **<0.0001** | 1 | 115.9 | **<0.0001** |
| Longitude | 1 | 4.77 | **0.02** | 1 | 9.78 | **0.002** | 1 | 0.11 | 0.73 | 1 | 11.76 | **0.0006** |
| Annual Precip. | 1 | 5.00 | **0.03** | 1 | 24.29 | **<0.0001** | 1 | 12.97 | **0.0003** | 1 | 72.9 | **<0.0001** |
| MAT | 1 | 0.71 | 0.39 | 1 | 8.32 | **0.004** | 1 | 15.19 | **<0.0001** | 1 | 24.02 | **<0.0001** |
| Soil C | 1 | 3.74 | 0.053 | 1 | 0.56 | 0.45 | 1 | 4.39 | **0.03** | 1 | 2.23 | 0.13 |
| Soil N | 1 | 0.05 | 0.81 | 1 | 4.34 | **0.037** | 1 | 12.96 | **0.0003** | 1 | 0.85 | 0.35 |
| Soil pH | 1 | 2.82 | 0.093 | 1 | 0.939 | 0.33 | 1 | 0.14 | 0.71 | 1 | 9.9 | **0.002** |
| DBH (F) | 1 | 0.004 | 0.95 | 1 | 1.85 | 0.17 | 1 | 7.21 | **0.007** | 1 | 0.03 | 0.86 |
| Foliar C:N (F) | 1 | 13.72 | **0.0002** | 1 | 0.0004 | 0.98 | 1 | 24.7 | **<0.0001** | 1 | 0.21 | 0.64 |
| Bud Break (GH) | 1 | 4.77 | **0.03** | 1 | 0.26 | 0.61 | 1 | 12.6 | **0.0003** | 1 | 1.19 | 0.27 |

**Supplemental Table 5. Relative importance of predictor variables for soil microbiome diversity**. Relative importance determined by summing the coefficients of the I-splines from GDM models. The most important predictor for tree and interspace soil bacterial and soil fungal communities is shown in bold. Predictors found to be not significant are indicated by dashes. (GH) denotes greenhouse-measured, genetically-based plant trait variation; (F) denotes field-measured plant traits. All 15 populations represented in Figure 2d & e are included in distance-based redundancy analysis. This table is visually represented in Figure 4.

| **Gradient** | **Tree Bacterial Community Dissimilarity** | **IS Bacterial Community Dissimilarity** | **Tree Fungal Community Dissimilarity** | **IS Fungal Community Dissimilarity** |
| --- | --- | --- | --- | --- |
| Annual Precipitation | 0.022 | **0.511** | 0.182 | 0.29 |
| Bud Break (GH) | 0.10 | -- | 0.09 | -- |
| DBH (F) | **0.29** | -- | 0.124 | -- |
| Elevation | -- | 0.053 | 0.023 | 0.188 |
| Foliar C:N (F) | 0.21 | -- | 0.104 | -- |
| Geographic Location | 0.116 | 0.133 | **0.656** | 0.56 |
| Mean Ann. Temp. | 0.234 | 0.141 | 0.255 | 0.262 |
| Specific Leaf Area (F) | 0.002 | -- | 0.11 | -- |
| Soil C | 0.191 | 0.04 | -- | 0.121 |
| Soil N | 0.182 | 0.36 | 0.11 | **0.61** |
| Soil pH | 0.22 | 0.117 | 0.12 | 0.058 |

**Supplemental Table 6.** Site characteristics of populations sampled across the range of *Populus angustifolia*. Climatic and edaphic characteristics are represented by mean population-level values for each river sampled. The 10 populations are a subset of 17 populations surveyed in 2012 (see^1^). Soil type was extracted from GIS layers published in Zobler (1999)^2^. MAT represents mean annual temperature of surveyed populations in degrees Celsius. AP represents annual precipitation of surveyed populations in centimeters (cm). Mean population-level total soil carbon (C) and nitrogen (N) are displayed as percentages.

| **River** | **Latitude** | **MAT (°C)** | **AP (cm)** | **Soil C (%)** | **Soil N (%)** | **Soil Type** |
| --- | --- | --- | --- | --- | --- | --- |
| **“Warm” populations** |  |  |  |  |  |  |
| Oak Creek, AZ | 35.1435 | 9.4 | 57.0 | 2.99 | 0.18 | Luvic Kastanozem |
| Blue River, AZ | 33.6677 | 8.8 | 47.2 | 1.47 | 0.09 | Luvic Kastanozem |
| Indian Creek, UT | 37.9460 | 8.1 | 37.5 | 2.52 | 0.16 | Calcic Yermosol |
| Lexington Creek, NV | 38.8604 | 7.1 | 35.4 | 7.01 | 0.29 | Luvic Yermosol |
| Snake Creek, NV | 38.9212 | 7.1 | 34.8 | 7.39 | 0.28 | Luvic Yermosol |
| **“Cool” populations** |  |  |  |  |  |  |
| Dolores River, CO | 37.6713 | 3.2 | 66.8 | 3.42 | 0.22 | Eutric Regosol |
| Shoshone River, WY | 44.4365 | 3.0 | 45.9 | 3.19 | 0.18 | Albic Luvisol |
| Rio Grande, CO | 37.5748 | 2.0 | 60.6 | 3.48 | 0.20 | Albic Luvisol |
| Snake River, WY | 43.5855 | 1.9 | 51.4 | 3.63 | 0.17 | Albic Luvisol |
| Gros Ventre River, WY | 43.5884 | 0.9 | 51.1 | 6.36 | 0.29 | Albic Luvisol |

**Supplemental Table 7.** Generalized linear and linear mixed effects model results exploring potential patterns of local adaptation in bud break phenology to ***live*** soil inoculation treatments. Separate models explore potential local adaptation in ***home vs away*** soil treatments, across soil inoculations from different populations, and across soil climatic origins (i.e., warm and cool). Significant predictors of experimental differences in bud break phenology are in bold. Bonferroni corrections were applied to account for multiple comparisons (*n*=4) and alpha value of 0.05 was divided by 4, corrected alpha=0.0125.

| ***Response***: Julian Day |  |  | *α=0.0125* |
| --- | --- | --- | --- |
| **Factors** generalized linear model / glm() | **DF** | ***X*^2^** | **Pr(>*X*^2^)** |
| Population | 9 | 64.911 | **<0.0001** |
| Soil source population | 10 | 12.44 | 0.256 |
| Population * Soil source population | 42 | 31.57 | 0.88 |
| ***Response***: Julian Day |  |  |  |
| **Factors** generalized linear model / glm() |  |  |  |
| Population | 9 | 68.6 | **<0.0001** |
| Home/Away | 1 | 0.071 | 0.789 |
| Population * Home/Away | 7 | 1.24 | 0.99 |
| ***Response***: Julian Day |  |  |  |
| **Factors** generalized linear model / glm() |  |  |  |
| Population | 9 | 66.7 | **<0.0001** |
| Soil source climatic origin | 1 | 4..67 | 0.03* |
| Population * Soil source climatic origin | 9 | 6.76 | 0.66 |
| ***Response***: Julian Day |  |  |  |
| **Factors** in linear mixed effects model / lmer() |  |  |  |
| Population climatic origin | 1 | 0.41 | 0.53 |
| Soil source climatic origin | 1 | 0.95 | 0.33 |
| Population climatic origin * Soil source climatic origin | 1 | 0.47 | 0.49 |

**Supplemental Table 8.** Generalized linear and linear mixed effects model results exploring potential patterns of local adaptation in bud break phenology to ***sterile*** soil inoculation treatments. Separate models explore potential local adaptation in ***home vs away*** soil treatments, across soil inoculations from different populations, and across soil climatic origins (i.e., warm and cool). Significant predictors of experimental differences in bud break phenology are in bold. Bonferroni corrections were applied to account for multiple comparisons (*n*=4) and alpha value of 0.05 was divided by 4, corrected alpha=0.0125.

| ***Response***: Julian Day |  |  | *α=0.0125* |
| --- | --- | --- | --- |
| **Factors** generalized linear model / glm() | **DF** | ***X*^2^** | **Pr(>*X*^2^)** |
| Population | 9 | 64.911 | **<0.0001** |
| Soil source population | 10 | 12.44 | 0.34 |
| Population * Soil source population | 38 | 31.57 | 0.96 |
| ***Response***: Julian Day |  |  |  |
| **Factors** generalized linear model / glm() |  |  |  |
| Population | 9 | 68.6 | **<0.0001** |
| Home/Away | 1 | 0.071 | 0.243 |
| Population * Home/Away | 6 | 1.24 | 0.446 |
| ***Response***: Julian Day |  |  |  |
| **Factors** generalized linear model / glm() |  |  |  |
| Population | 9 | 66.7 | **<0.0001** |
| Soil source climatic origin | 1 | 4.67 | *0.076* |
| Population * Soil source climatic origin | 8 | 6.76 | 0.59 |
| ***Response***: Julian Day |  |  |  |
| **Factors** in linear mixed effects model / lmer() |  |  |  |
| Population climatic origin | 1 | 1.21 | 0.27 |
| Soil source climatic origin | 1 | 3.76 | *0.05* |
| Population climatic origin * Soil source climatic origin | 1 | 0.76 | 0.38 |

**Supplemental Table 9. Soil microbiomes mediate plant phenology.** Linear mixed effects model results exploring the response of bud break phenology (Julian days) to soil inoculation treatments (results represented in Figure 5b). Separate reduced models were run to explore significant interactive effect in full model. Reduced models included: 1) live/sterile as a fixed effect and population as a random effect for both warm and cool soil origins; and 2) Soil source climatic origin as a fixed effect and population as a random effect for both live and sterile soil inoculation treatments. Significant predictors of experimental differences in bud break phenology are in bold.

| ***Response****: Julian Day (full model)* |  |  |  |
| --- | --- | --- | --- |
| **Factors** | **DF** | ***X*^2^** | **Pr(>*X*^2^)** |
| Live/Sterile | 1 | 4.621 | **0.031** |
| Soil source climatic origin | 1 | 6.820 | **0.009** |
| Live/Sterile * Soil source climatic origin | 1 | 9.6765 | **0.001** |
| ***Response****: Julian Day (within warm climatic origin)* |  |  |  |
| **Factors** | **DF** | ***X*^2^** | **Pr(>*X*^2^)** |
| Live/Sterile | 1 | 5.190 | **0.039** |
| ***Response****: Julian Day (within cool climatic origin)* |  |  |  |
| **Factors** | **DF** | ***X*^2^** | **Pr(>*X*^2^)** |
| Live/Sterile | 1 | 5.415 | **0.019** |
| ***Response****: Julian Day (within live inoculated)* |  |  |  |
| **Factors** | **DF** | ***X*^2^** | **Pr(>*X*^2^)** |
| Soil Source climatic origin | 1 | 7.556 | **0.005** |
| ***Response****: Julian Day (within sterile inoculated)* |  |  |  |
| **Factors** | **DF** | ***X*^2^** | **Pr(>*X*^2^)** |
| Soil Source climatic origin | 1 | 2.72 | 0.11 |

**Supplemental Table 10. Biotic interaction Temperature Distance (Δ°C) is related to plant phenology.** Linear mixed effects model results exploring the relationship between Δ°C and bud break phenology (Julian days). “Temperature transfer” represents the difference in the tree’s climatic origin and the soil inoculum’s climatic origin (i.e., Δ°C). Live/Sterile delineates between live or sterile inocula and directly tests the effect of having microbes present (live) or absent (sterile). Significant predictors are in bold.

| ***Response****: Julian Day (full model)* |  |  |  |
| --- | --- | --- | --- |
| **Factors** | **DF** | ***X*^2^** | **Pr(>*X*^2^)** |
| Temperature transfer (Δ°C) | 1 | 5.761 | **0.016** |
| Live/Sterile | 1 | 0.0001 | 0.992 |
| Temperature transfer (Δ°C) * Live/Sterile | 1 | 10.775 | **0.0010** |
| ***Response****: Julian Day (within live inoculated)* |  |  |  |
| **Factors** | **DF** | ***X*^2^** | **Pr(>*X*^2^)** |
| Temperature transfer (Δ°C) | 1 | 7.257 | **0.007** |
| ***Response****: Julian Day (within sterile inoculated)* |  |  |  |
| **Factors** | **DF** | ***X*^2^** | **Pr(>*X*^2^)** |
| Temperature transfer (Δ°C) | 1 | 2.631 | 0.11 |

**References**

1. I.M. Ware *et al.*, Climate-driven reduction of genetic variation in plant phenology alters soil

communities and nutrient pools. *Global Change Bio.* **25,** 1514-1528 (2019).

2. L. Zobler, Global Soil Types, 1-Degree Grid (Zobler). Data set. Available

on-line [[http://www.daac.ornl.gov](http://www.daac.ornl.gov/)] from Oak Ridge National Laboratory Distributed Active Archive Center, Oak Ridge, Tennessee, U.S.A. [doi:10.3334/ORNLDAAC/418](http://dx.doi.org/10.3334/ORNLDAAC/418) (1999).
