# Supplementary material for: Climate-driven divergence in plant-microbiome interactions generates range-wide variation in bud break phenology
Source: Commun Biol. 2021 Jun 16;4:748. doi: 10.1038/s42003-021-02244-5 (PMC8209103; doi:10.1038/s42003-021-02244-5)
Supplement: Supplementary file 2 — Description of Additional Supplementary Files [file 42003_2021_2244_MOESM2_ESM.pdf]

Description of Additional Supplementary Files

File name: Supplemental Data 1

Description: Full 16S Taxonomy: 16S_ASV_table_taxa_filtered.csv

File name: Supplemental Data 2

Description: Full ITS Taxonomy: ITS_SAV_table_taxa_filtered.csv

File name: Supplemental Data 3

Description: Bacterial Indicator Taxa List: bac_ISA_output_tree.csv

File name: Supplemental Data 4

Description: Fungal Indicator Taxa List with FUNGuild results from Warm Climatic Origin: Warm_fun_tree_ISA_guilds.csv

File name: Supplemental Data 5

Description: Fungal Indicator Taxa List with FUNGuild results from Cool Climate Origin: Cool_fun_tree_ISA_guilds.csv

File name: Supplemental Data 6

Description: Bacterial community turnover dataset (Figure 2): 16S_turnover.csv

File name: Supplemental Data 7

Description: Fungal community turnover dataset (Figure 2): ITS_turnover.csv

File name: Supplemental Data 8

Description: Mean differences in soil chemistry between tree and interspace soil samples (Figure 2): soil_chem_turnover.csv

File name: Supplemental Data 9

Description: Standardized splines for tree associated, bacterial community GDMs (Figure 3): prok_tree_splines_std.csv

File name: Supplemental Data 10

Description: Standardized splines for interspace, bacterial community GDMs (Figure 3): prok_is_splines_std.csv

File name: Supplemental Data 11

Description: Standardized splines for tree associated, fungal community GDMs (Figure 3): fung_tree_splines_std.csv

File name: Supplemental Data 12

Description: Standardized splines for interspace, fungal community GDMs (Figure 3): fung_is_splines_std.csv

File name: Supplemental Data 13

Description: Data generated from soil inoculation experiment (Figure 4): soil_inoculation_experiment.csv
