# Supplementary material for: Climate-driven divergence in plant-microbiome interactions generates range-wide variation in bud break phenology
Source: Commun Biol. 2021 Jun 16;4:748. doi: 10.1038/s42003-021-02244-5 (PMC8209103; doi:10.1038/s42003-021-02244-5)
Supplement: Supplementary file 16 — Reporting Summary [file 42003_2021_2244_MOESM16_ESM.pdf]

nature research

Corresponding author(s): Dr. Ian M. Ware

Last updated by author(s): 3/272021

Reporting Summary

Nature Research wishes to improve the reproducibility of the work that we publish. This form provides structure for consistency and transparency in reporting. For further information on Nature Research policies, see our Editorial Policies and the Editorial Policy Checklist.

Statistics

For all statistical analyses, confirm that the following items are present in the figure legend, table legend, main text, or Methods section.

n/a	Confirmed	
[ ]	[x]	The exact sample size (*n*) for each experimental group/condition, given as a discrete number and unit of measurement
[ ]	[x]	A statement on whether measurements were taken from distinct samples or whether the same sample was measured repeatedly
[ ]	[x]	The statistical test(s) used AND whether they are one- or two-sided<br>*Only common tests should be described solely by name; describe more complex techniques in the Methods section.*
[ ]	[x]	A description of all covariates tested
[ ]	[x]	A description of any assumptions or corrections, such as tests of normality and adjustment for multiple comparisons
[ ]	[x]	A full description of the statistical parameters including central tendency (e.g. means) or other basic estimates (e.g. regression coefficient) AND variation (e.g. standard deviation) or associated estimates of uncertainty (e.g. confidence intervals)
[ ]	[x]	For null hypothesis testing, the test statistic (e.g. *F*, *t*, *r*) with confidence intervals, effect sizes, degrees of freedom and *P* value noted<br>*Give P values as exact values whenever suitable.*
[x]	[ ]	For Bayesian analysis, information on the choice of priors and Markov chain Monte Carlo settings
[x]	[ ]	For hierarchical and complex designs, identification of the appropriate level for tests and full reporting of outcomes
[x]	[ ]	Estimates of effect sizes (e.g. Cohen's *d*, Pearson's *r*), indicating how they were calculated

Our web collection on statistics for biologists contains articles on many of the points above.

Software and code

Policy information about availability of computer code

Data collection	Open source code and software was used for data collection. BBTools was used to trim, fillter and merge microbial DNA samples. DADA2 version 1.6.0 was used to identify amplicon sequence variants. Lastly, FUNGuild is an open annotation tool and was used to assign functional guilds to fungal taxa lists.
Data analysis	Open source code was used in the data analysis. All open source was performed in using R: The R project for Statistical Computing version 3.5.3. All requests for code availability can be addressed to IMW.

For manuscripts utilizing custom algorithms or software that are central to the research but not yet described in published literature, software must be made available to editors and reviewers. We strongly encourage code deposition in a community repository (e.g. GitHub). See the Nature Research guidelines for submitting code & software for further information.

Data

Policy information about availability of data

All manuscripts must include a data availability statement. This statement should provide the following information, where applicable:

- Accession codes, unique identifiers, or web links for publicly available datasets
- A list of figures that have associated raw data
- A description of any restrictions on data availability

Amplicon sequences are archived in the National Center for Biotechnology Information SRA database (BioProject accession number: PRJNA726831). Observational and experimental data generated during and/or analyzed during the current study can be found as Supplementary Data 1-13.

Field-specific reporting

Please select the one below that is the best fit for your research. If you are not sure, read the appropriate sections before making your selection.

☐ Life sciences ☐ Behavioural & social sciences ☒ Ecological, evolutionary & environmental sciences

For a reference copy of the document with all sections, see nature.com/documents/nr-reporting-summary-flat.pdf

Ecological, evolutionary & environmental sciences study design

All studies must disclose on these points even when the disclosure is negative.

Study description	Soil microbiomes are rapidly becoming known as an important driver of variation in plant phenotypes and may mediate plant responses to environmental factors, including warming and drought. However, integrating spatial scales relevant to climate change with plant intraspecific genetic variation and soil microbial ecology is difficult, making studies of broad inference rare. Here we hypothesize and show: 1) the degree to which tree genotypes condition their soil microbiomes varies by population across the geographic distribution of a widespread riparian tree, Populus angustifolia; 2) geographic dissimilarity in soil microbiomes among populations is influenced by both abiotic and biotic environmental variation; and 3) soil microbiomes that vary in response to abiotic and biotic factors can change plant foliar phenology. We show that soil microbiomes respond to intraspecific variation at the tree genotype and population level, and geographic variation in soil characteristics and climate. Using a fully reciprocal plant population by soil location feedback experiment, we identified a climate-based soil microbiome effect that advanced and delayed bud break phenology by approximately 10 days overall. Geographic functional variation in the soil microbial community mediates variation in foliar bud break phenology. These results demonstrate a landscape-level feedback between tree populations and their associated soil microbial communities and suggest that soil microbes may play important roles in mediating and buffering bud break phenology with climate warming, with whole ecosystem implications.
Research sample	Populus angustifolia James is a dominant tree species distributed throughout high elevation riparian zones (900 to 2500 m) along the Rocky Mountains from southern Alberta, through the intermountain United States, and into northern Mexico. During May and June 2012, 17 distinct P. angustifolia populations were surveyed collectively from three different genetic provenances (Arizona, Eastern, and Northern/Wasatch Clusters) across a gradient of ~1700 kilometers of latitude from southeastern Arizona to south central Montana.
Sampling strategy	Field sampling at each tree population was performed random in nature to maximum variation in tree phenotypes and the soil environment. This work spanned 17 populations and 3 underlying genetic provenances of Populus angustifolia. Each tree population has multiple sites spanning the elevational extent of Populus angustifolia in that riparian corridor. Great effort was taken to maximize sampling size and general feasibility of the study.
Data collection	Field data collections were performed in two seperate years: 2012 and 2015. Field collections in 2012 were performed by Ian Ware, Michael Van Nuland, Courtney Gorman, Jennifer Schweitzer, and Joe Bailey. Field collections in 2015 were performed by Ian Ware. To separate the conditioning effects of P. angustifolia from underlying site differences, tree-conditioned soils were collected at the base of each trunk (within 0.25 m) and unconditioned interspace soils were collected from a random location away from the tree canopy, no less than five meters from the trunk and consistently outside of the drip line of each tree canopy. To capture the range of genetic variation that occurred in each tree population, we identified and sampled from 3 to 5 collection sites within each population: the highest and lowest elevation site with P. angustifolia trees and variable intermediate locations (1–3) within each riparian area.Experimental data collection spanned 2015-2017 and was performed by Ian Ware, Liam Mueller, Michael Van Nuland. Mean characteristics measured in the field and inoculation study did not statistically vary by data collector.
Timing and spatial scale	Field data collections were performed in two separate years: May - June 2012 and May - June 2015. Seventeen distinct P. angustifolia populations were surveyed collectively from three different genetic provenances (Arizona, Eastern, and Northern/Wasatch Clusters) across a gradient of ~1700 kilometers of latitude from southeastern Arizona to south central Montana. Tree populations were sampled after spring bud break and leaf flush, and sampling efforts starting in the most southern site and headed northward until all populations were sampled.
Data exclusions	No data was excluded
Reproducibility	Each individual detail of sampling effort, experimental design, data processing, and statistical approach are included in the Methods section to best ensure reproducibility of this study.
Randomization	After soil inoculations, transplanted cuttings were randomized using a random number generator on the bench tops to remove any microsite variation in light and temperature within the greenhouse.
Blinding	Blinding was not needing in this study as trees were not aware of which soil inoculation they were to receive.

Did the study involve field work? ☒ Yes ☐ No

Field work, collection and transport

Field conditions	The study conditions for field collections were indicitive of late spring and early summer in the Rocky Mountain Region of the United States. 95% of sampling days were sunny ranging in temperature from 12 - 29 degrees C.

Location	The sampling occurred across 17 tree populations. Below are the mean latitude and longitude for each sampled tree population.<br>Oak Creek, Arizona – Latitude: 34.990776, Longitude: -111.74257<br>Blue River, Arizona – Latitude: 33.593919, Longitude: -109.14107<br>GSD, Colorado – Latitude: 37.756176, Longitude: -105.502358<br>Indian Creek, Utah – Latitude: 38.025365, Longitude: -109.540135<br>Park Creek, Colorado – Latitude: 37.592869, Longitude: -106.729209<br>San Juan River, Colorado – Latitude: 37.679719, Longitude: -106.60324<br>Dolores River, Colorado – Latitude: 37.848339, Longitude: -107.882816<br>San Miguel River, Colorado – Latitude: 38.266464, Longitude: -108.401136<br>Snake Creek, Nevada – Latitude: 38.95, Longitude: -114.129518<br>Lexington Creek, Nevada – Latitude: 38.6, Longitude: -114.179945<br>Ogden River, Utah – Latitude: 41.2798, Longitude: -111.65629<br>Weber River, Utah – Latitude: 41.136263, Longitude: -111.90438<br>Logan River, Utah – Latitude: 41.863977, Longitude: -111.571419<br>Snake River, WY – Latitude: 43.821814, Longitude: -110.300298<br>Gros Ventre River, WY – Latitude: 43.552321, Latitude: -110.280754<br>Shoshone River, WY – Latitude: 43.178643, Longitude: -110.984307<br>Yellowstone River, MT – Latitude: 45.634923, Longitude: -110.571109
Access & import/export	Sampling sites were accessed by foot and treated in a responsible manner, minimizing any negative impacts during access.
Disturbance	Any potential disturbances from soil collections was limited due to the small volume of soil collection.

Reporting for specific materials, systems and methods

We require information from authors about some types of materials, experimental systems and methods used in many studies. Here, indicate whether each material, system or method listed is relevant to your study. If you are not sure if a list item applies to your research, read the appropriate section before selecting a response.

Materials & experimental systems

n/a	Involved in the study
☒	☐ Antibodies
☒	☐ Eukaryotic cell lines
☒	☐ Palaeontology and archaeology
☐	☒ Animals and other organisms
☒	☐ Human research participants
☒	☐ Clinical data
☒	☐ Dual use research of concern

Methods

n/a	Involved in the study
☒	☐ ChIP-seq
☒	☐ Flow cytometry
☒	☐ MRI-based neuroimaging

Animals and other organisms

Policy information about studies involving animals; ARRIVE guidelines recommended for reporting animal research

Laboratory animals	NA
Wild animals	NA
Field-collected samples	Live soil inocula were collected. In 2012, soil samples were collected with a 2.5 cm diameter Oatfield soil core to a vertical depth of 15 cm, placed in a plastic bag, transported cold from the field and stored at 4°C in the lab until analysis. Field collected wet soils were sieved to 2 mm and then sub-sampled and preserved for various analyses (see 25). Soil DNA was extracted from a 0.25 g frozen sub-sample of each sieved soil by using the Power Soil DNA isolation kit (MoBio, Carlsbad, CA USA) according to the manufacturer's instructions. Quantitative PCR reactions to assess bacterial and fungal abundance in each field soil sample were performed after in 96-well plates on a CFX96 real-time PCR detection system (Bio-Rad Laboratories, Hercules, CA USA). Samples were amplified for the 16S v4 region using primers 515F/806R, and for the ITS2 region using primers ITS9F/ITS4R from a subset of the total field collections (~270 samples; across 15 populations). Samples were sent to the Department of Energy Joint Genome Institute for sequencing on an Illumina MiSeq (2x300bp; Illumina Inc., San Diego, CA).
Ethics oversight	NA

Note that full information on the approval of the study protocol must also be provided in the manuscript.
